# The N-Terminal Extension Domain of the *C. elegans* Half-Molecule ABC Transporter, HMT-1, Is Required for Protein-Protein Interactions and Function

**DOI:** 10.1371/journal.pone.0012938

**Published:** 2010-09-23

**Authors:** Sungjin Kim, Devarshi S. Selote, Olena K. Vatamaniuk

**Affiliations:** Department of Crop and Soil Sciences, Cornell University, Ithaca, New York, United States of America; Indiana University, United States of America

## Abstract

**Background:**

Members of the HMT-1 (**h**eavy **m**etal **t**olerance factor **1**) subfamily of the ATP-binding cassette (ABC) transporter superfamily detoxify heavy metals and have unique topology: they are half-molecule ABC transporters that, in addition to a single transmembrane domain (TMD1) and a single nucleotide-binding domain (NBD1), possess a hydrophobic NH2-terminal extension (NTE). These structural features distinguish HMTs from other ABC transporters in different species including *Drosophila* and humans. Functional ABC transporters, however, are comprised of at least four-domains (two TMDs and two NDBs) formed from either a single polypeptide or by the association of two or four separate subunits. Whether HMTs act as oligomers and what role the NTE domain plays in their function have not been determined.

**Methodology/Principal Findings:**

In this study, we examined the oligomeric status of *Caenorhabditis elegans* HMT-1 and the functional significance of its NTE using gel-filtration chromatography in combination with the mating-based split-ubiquitin yeast two-hybrid system (mbSUS) and functional *in vivo* assays. We found that HMT-1 exists in a protein complex in *C. elegans*. Studies in *S. cerevisiae* showed that HMT-1 at a minimum homodimerizes and that oligomerization is essential for HMT-1 to confer cadmium tolerance. We also established that the NTE domain plays an important structural and functional role: it is essential for HMT-1 oligomerization and Cd-detoxification function. However, the NTE itself was not sufficient for oligomerization suggesting that multiple structural features of HMT-1 must associate to form a functional transporter.

**Conclusions:**

The prominence of heavy metals as environmental toxins and the remarkable conservation of HMT-1 structural architecture and function in different species reinforce the value of continued studies of HMT-1 in model systems for identifying functional domains in HMT1 of humans.

## Introduction

Increasing emission of heavy metals and metalloids such as cadmium (Cd), mercury (Hg), lead (Pb) and arsenic (As) into food, water and air poses major health and environmental problems. Members of the **h**eavy **m**etal **t**olerance factor 1 (HMT-1) transporter family are acutely required for detoxification of heavy metals and belong to the “B” branch of the ATP-bindings cassette (ABC) transporters superfamily [1]–[7]. It has been proposed that HMTs transport heavy metals coordinated to glutathione (GSH) derivatives named phytochelatins (PC) [8]. Recent genetic and biochemical studies have shown that HMTs act independently of PC, but how they function and their physiological substrates are not known [2], [7], [9], [10].

HMT-1 proteins share a conserved architecture that distinguishes them from other ABC transporters in diverse species including *Schizosaccharomyces pombe*, *Chlamydomonas reinhardtii*, *Caenorhabditis elegans*, *Drosophila melanogaster*, *Rattus norvegicus*, and *Homo sapiens* [2], [4], [5], [9], [11]. HMTs are half-molecule ABC transporters containing one polytopic membrane domain (TMD1) and one ATP-binding domain (NBD1) and are the only half-transporters that, in addition to TMD1 and NBD1, possess a hydrophobic NH2-terminal extension (NTE) [2], [3], [12]. Based on solved crystal structures of ABC transporters from prokaryotes, formation of at least a four-domain structure (two TMDs and two NBDs) is a prerequisite to mediate the Mg·ATP-powered translocation of substances across a lipid bilayer [13]. The four-domain structure of ABC transporters can be formed from a single polypeptide or by the association of two or four separate subunits [14]. In eukaryotes, most ABC proteins are encoded as single polypeptides containing two TMDs and two NBDs [15]. In contrast, half-molecule ABC transporters function by forming homo- or heterooligomers and/or complexes with other cellular components [15]–[20]. For instance, the peroxisomal half-transporter ALDP (ABCD1) can homodimerize or heterodimerize with related ABC half-transporters ALDPR (ABCD2) or PMP70 (ABCD3) and interfering with dimerization disrupts ALDP function [18]. Mammalian half-transporters TAP1 and TAP2 form heterodimers to transport peptide degradation products from the cytosol into the lumen of the endoplasmic reticulum [16], [19], [20]. The sterol half-transporters ABCG5 and ABCG8 must heterodimerize in order to get to the cell surface [21]. Interestingly, some full-molecule ABC transporters oligomerize as well: for instance, human ABCC1/MRP1, with two TMDs, two NBDs and an NTE, forms functional homodimer and homodimerization is regulated through the NTE domain [22].

Whether HMTs form higher order complexes and the role of their NTE domain have not been investigated. Because *C. elegans* HMT-1 (CeHMT-1) is expressed in liver-like cells, the coelomocytes, as well as head neurons and intestinal cells, which are the cell types that are affected by heavy metal poisoning in humans [7], we have used *C. elegans* as a model system for identifying functional domains of HMT-1 expecting that our studies will provide insights into the function of equivalent domains in HMTs in higher animals.

We now show that HMT-1 exists as an oligomer *in vivo*, can self-associate in yeast and that oligomerization is required for the ability of HMT-1 to detoxify cadmium. We also show that the NTE domain is essential for HMT-1 self-association and function. However, unlike the NTE of ABCC1/MRP1, the NTE of HMT-1 is not sufficient for self-association suggesting that multiple regions of HMT-1 must associate with one another to form an active transporter.

## Results and Discussion

### HMT-1 Exists in a Protein Complex in *C. elegans*

**Figure 1:**
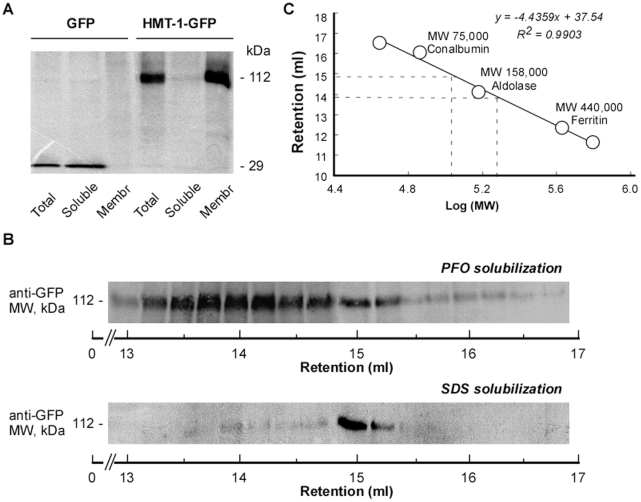
HMT-1 forms oligomeric complexes. **A.** SDS/PAGE and Western blot analyses of HMT-1::GFP. Aliquots (30 µg/lane) of total (*Total*), soluble (*Soluble*) or microsomal membrane proteins (*Membr*) isolated from worms expressing either HMT-1::GFP (*HMT-1-GFP*) or GFP alone (*GFP*) under the control of the *hmt-1* promoter were subjected to SDS-PAGE and immunoblot analysis. **B.** Membrane proteins isolated from HMT-1::GFP expressing worms were solubilized either with PFO (*PFO-solubilization*) or with SDS (*SDS-solubilization*) and separated by FPLC on Superose 6HR column. Fractions (250 µl) were collected, proteins were precipitated with TCA, and HMT-1::GFP was detected by SDS/PAGE and Western analysis. **C.** The apparent molecular masses of PFO- or SDS- extracted HMT-1::GFP after FPLC analysis were estimated based on the linear regression of the retention time of molecular mass markers.

We first sought to test whether HMT-1 exists as an oligomer in *C. elegans*. Towards this goal, we generated transgenic worms expressing functional translational HMT-1::GFP fusions and analyzed the HMT-1::GFP in fractioned worm lysates. As would be expected for integral membrane protein, SDS-PAGE and immunoblot analyses of fractionated lysates from *phmt-1-hmt-1::GFP* worms identified HMT-1::GFP among total and membrane proteins, but not among soluble proteins (Fig. 1A). We also fractioned transgenic worms that express transcriptional *phmt-1::GFP* fusions. Since the GFP polypeptide does not possess membrane-spanning domains, it localized only in the total and soluble, but not in the membrane fraction of proteins (Fig. 1A).

We next performed a gel-filtration separation of HMT-1::GFP by fast protein liquid chromatography (FPLC) on a Superose 6HR column (GE Healthcare). We solubilized HMT-1::GFP with perfluorooctanoate (PFO), a mild detergent that preserves interactions between protein subunits and has been successfully used in studies of ABC transporters [23], [24]. We also extracted HMT-1::GFP with SDS, a strong ionic detergent, which disrupts protein interactions [25]. If HMT-1::GFP exists as a monomer *in vivo*, PFO- and SDS-extracted HMT-1::GFP would have identical migration properties. However, if HMT-1::GFP forms higher oligomeric states, the migration properties would be distinct.

We established that the elution peak of SDS-extracted HMT-1::GFP was at 15 ml, corresponding to an estimated M_r_ of 125,000 (Fig. 1B, C). Since the calculated molecular mass of HMT-1::GFP is 117,000, the observed elution profile (Fig. 1B,C) is consistent with the migration of an HMT-1::GFP monomer. In contrast, PFO-extracted HMT-1::GFP was eluted as a broad peak at 13-15.25 ml (Fig. 1B, C). The highest the anti-GFP antibody immunoreactivity was observed in fractions 13.75–14.25 ml, corresponding to estimate molecular masses of 340–269 kDa (Fig. 1B, C). These estimated molecular masses suggest that HMT-1::GFP is present almost exclusively in a protein complex either with itself or/and with other proteins in *C. elegans*.

### Detection of HMT-1–HMT-1 Interactions Using Mating-Based Split-Ubiquitin Yeast-Two-Hybrid System (mbSUS)

**Figure 2:**
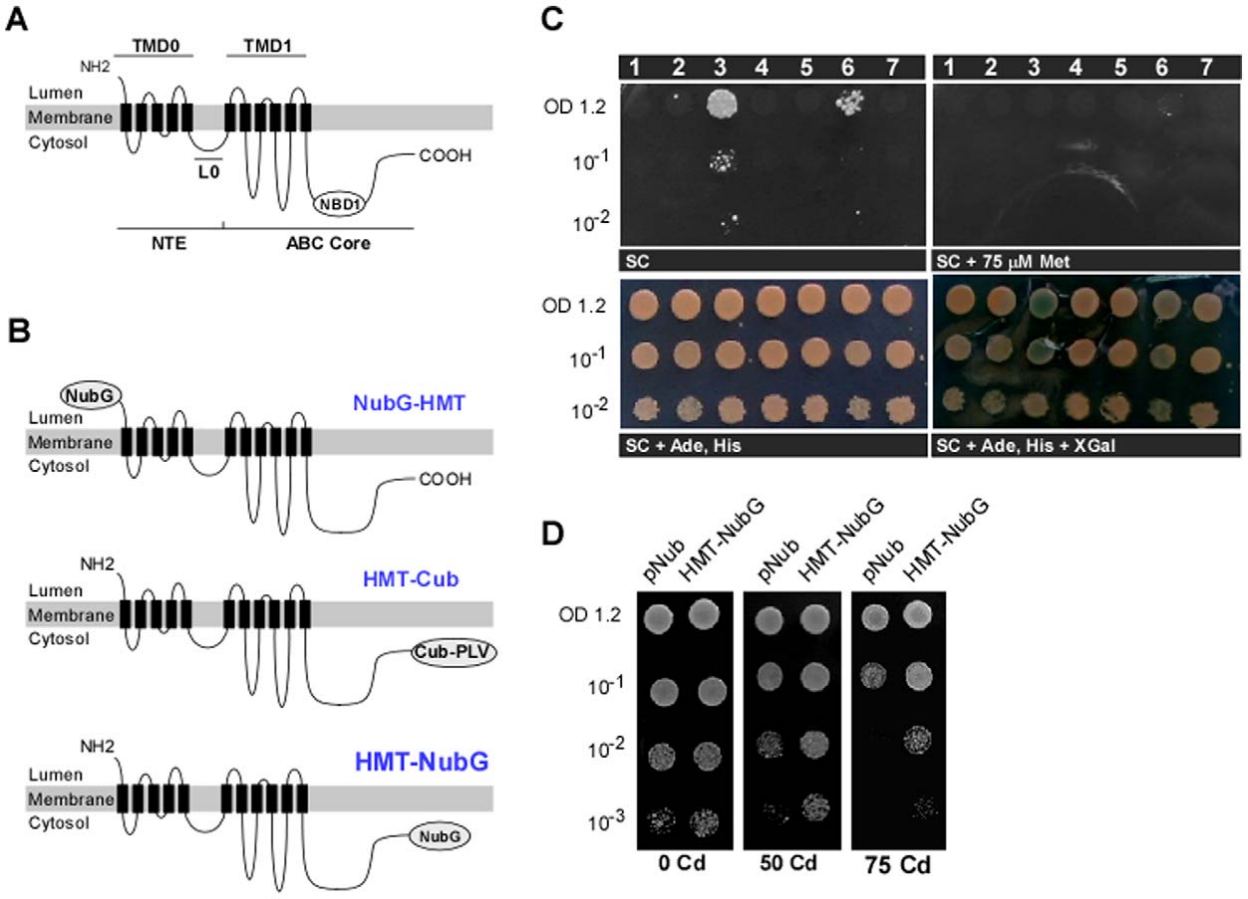
HMT-1 interacts with itself. **A.** Schematic representation of the topology of the full-length HMT-1 polypeptide. Based on the predicted topology (TMHMM software, version 2.0), the NH2-terminus is located outside (*Lumen*), whereas the COOH-terminus is inside (*Cytosol*). Also shown are the HMT-1 core region (*ABC core*) consisting of a single transmembrane domain (*TMD1*) with six transmembrane spans and a single nucleotide binding domain (*NBD1*). In addition to a core region, HMT-1 possesses the N-terminal extension (*NTE*), comprised of a membrane spanning domain (*TMD0*) and a linker domain (*L0*). **B.** Full-length HMT-1 was fused at the C-terminus with CubPLV (*HMT-Cub*) or with NubG at the C- or N-termini (*NubG-HMT* and *HMT-NubG*, respectively). The orientation of CubPLV and NubG is based on the predicted topology of HMT-1. **C.** Protein-protein interactions of HMT-1 as detected by mbSUS. Growth conditions are indicated below each panel; concentrations of yeast cells are indicated on the left. Numbers across the top represent experiments and controls as follows. Interaction tests where HMT-1-CubPLV was used as bait: 2, HMT-Cub + NubG-HMT; 3, HMT-Cub + HMT-NubG; 7, HMT-Cub + KAT1-NubG. Controls for self-activation: 1, HMT-Cub + NubG; 4, Cub + HMT-NubG. Interaction assays using AtKAT1-CubPLV as bait: KAT1-Cub + HMT-NubG (negative control); 6, KAT1-Cub + KAT1-NubG (positive control, [27]); 7, HMT-Cub + KAT1-NubG (negative control). Shown are representative results of at least three biological replicates. (SC =  synthetic complete medium; Met  =  methionine; Ade =  adenine; His = histidine). **D.** HMT-1-Nub confers Cd tolerance. Serial dilutions of yeast expressing pNub or HMT-NubG were as indicated. Concentrations of CdCl_2_ in µM are indicated below each experiment. Note the striking growth difference at 75 µM.

**Figure 3:**
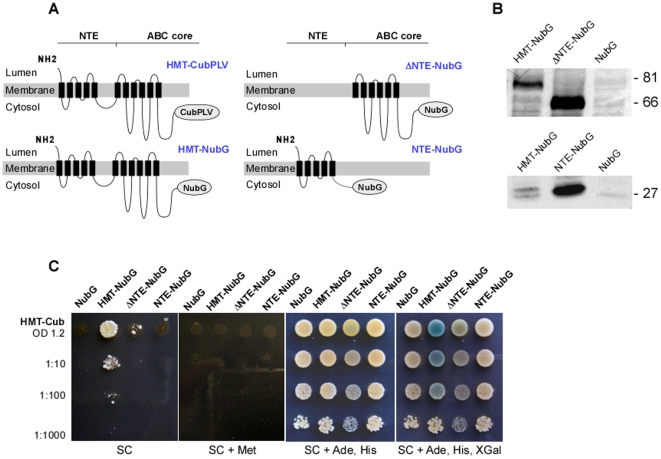
NTE is essential, but not sufficient for protein-protein interactions of HMT-1. **A.** Full-length HMT-1 was fused at the C-terminus with CubPLV (*HMT-Cub*) or with NubG (*HMT-NubG*). HMT-1 lacking NTE was fused at the C terminus with NubG (*ΔNTE-NubG*). **B.** SDS-PAGE and immunoblot analysis of microsomal membranes prepared from THY.AP5 cells expressing the NubG-fused full-length HMT-1 (*HMT-NubG*), NubG-fused HMT-1 lacking NTE (*ΔNTE-NubG*), NTE-fused to NubG (*NTE-NubG*), or empty NubG vector (*NubG*). **C**. Interactions were manifested by the ability of cells to grow on SC media without adenine and histidine (SC), but not in SC media with methionine (SC + Met). Minimal growth relative to the negative controls (co-expression with vector alone, *NubG*) was observed when HMT-1-NubG prey construct lacking NTE (*ΔNTE-NubG*) was co-expressed with the full-length HMT-1-CubPLV bait (*HMT-Cub*). Diploid cells did not grow when the NTE domain fused to NubG (NTE-NubG) was co-expressed with HMT-1-CubPLV bait (*HMT-Cub*). Interactions were also visualized by the presence of *β*-galactosidase activity. Note the presence of *β*-galactosidase activity in cells co-expressing the full-length HMT-1-NubG and HMT-1-CubPLV, but not the full-length HMT-1-CubPLV with ΔNTE-NubG, or NTE-NubG or NubG only.

The simplest explanation of the gel-filtration data is that HMT-1 homomerizes. To test this we used a mbSUS that detects binary interactions of membrane proteins *in vivo* [26], [27]. To do so, different HMT-1 fusions with ubiquitin were constructed (Figs. 2A, B, 3A), and interactions were monitored by the release of the artificial transcriptional factor PLV that activated the expression of *lexA*-driven reporter genes, *ADE2, HIS3* and *lacZ*. Based on the predicted membrane topology (TMHMM software, version 2.0 (http://www.cbs.dtu.dk/services/TMHMM-2.0/), the HMT-1 NH2-terminus is located outside (*Lumen*), whereas the COOH-terminus is inside (*Cytosol*) (Fig. 2A). HMT-1 “bait” vector was generated by fusing the C-terminus of the full-length of HMT-1 with a CubPLV fusion peptide (Fig. 2B). Since interactions can be detected only when CubPLV fusion and NubG fusions are in the cytosol [26], [27], we generated two HMT-1 prey constructs by fusing NubG at NH2- or COOH-termini (“NubG-HMT-1” and “HMT-1-NubG,” respectively (Fig. 2B). Therefore, if the topology prediction is correct, NubG of HMT-1-NubG -prey fusions would be localized in the cytosol (Fig. 2B) and would promote detection of interactions. In contrast, NubG of the NubG-HMT-1-prey construct will be localized intra-organellarly that will prevent interaction read-out (Fig. 2B). This experimental design assessed the membrane topology of HMT-1 and provided a negative control for spurious interactions. For other controls we co-expressed the following constructs: for detecting false positives due to self-activation, HMT-1-NubG or HMT-1-CubPLV were co-expressed with the empty pMetYCgate or pXNgate vectors respectively; as a positive control of interactions, the potassium channel, KAT1, from *Arabidopsis thaliana*, was used (KAT1-Cub-PVL bait and KAT1-NubG prey [27]); finally KAT1 was used as bait or prey for showing the specificity of HMT-1 interactions. The interactions were visualized in diploid cells by their ability to grow on SC medium lacking adenine and histidine, and by β-galactosidase activity assays. We ascertained that interactions occur only due to expression of HMT-1-CubPLV, or in case of the positive control, KAT1-CubPLV, by suppressing their expression with methionine (Fig. 2C).

Our data show that regardless of whether interactions were monitored as colony formation on selective media or by β-galactosidase activity, interactions did not occur between HMT-1-CubPLV and NubG lacking the HMT-1 insert (Fig. 2C). As would be expected for membrane proteins participating in different biological processes, interactions did not occur between *C. elegans* HMT-1 and *A. thaliana* KAT-1, regardless of the vector combination used in the study (Fig. 2C). Furthermore, HMT-1-HMT-1 interactions were not detected when NubG was placed at the HMT-1 amino terminus. Instead, we detected interactions only when NubG was placed on the carboxyl terminus.

Based on these results we propose that: *first*, HMT-1 at a minimum can form homodimers; *second*, the COOH-terminus of HMT-1 localizes in the cytosol since interactions were detected only when CubPLV and NubG were fused at the C-termini. Our data also suggest that the NH2-terminus of HMT-1 may be in the lumen. However, it is also possible that the amino terminal fusion is on the same side of the membrane but is not accessible to the Cub-PLV bait. Additional studies will determine the precise topology of HMT-1.

### HMT-1 of *C. elegans* Increases Cd Tolerance of *S. cerevisiae*

We showed previously that HMT-1 functions independently of PC synthases in heavy metal detoxification [2], [7], [9]. Therefore, HMTs are expected to increase heavy metal tolerance of organisms that lack the capacity to produce PC. Consistent with this, Preveral and colleagues showed that SpHMT-1 increases heavy metal tolerance of *E. coli* and *S. cerevisiae*, whose genomes lack PC synthase homologs [10]. Here we tested whether CeHMT-1 is able to increase Cd tolerance of *S. cerevisiae*, and in doing so, test if the interacting construct, HMT-1-NubG, is functional. In this assay, we compared Cd sensitivity of THY.AP5 yeast expressing the empty pNXgate 32/33-3HA (NubG) *vs.* THY.AP5 expressing HMT-1-NubG.

We first established that THY.AP5 cells expressing the empty vector were sensitive to 50 µM CdCl_2_ and their sensitivity increased with increasing Cd concentration in the culture medium (Fig. 2D). In contrast, THY.AP5 cells expressing HMT-1-NubG were more tolerant to Cd and were able to grow at a concentration of Cd (75 µM), that blocked growth of yeast cells expressing vector without HMT-1 cDNA insert (Fig. 2D).

These findings show that: *first*, HMT-1-NubG construct is functional because it increases Cd tolerance in *S. cerevisiae*; *second*, the ability of CeHMT-1 to increase heavy metal tolerance in *S. cerevisiae*, whose genome lacks PC synthase homologs, complements our previous findings and observation of others that HMTs act independently of PC synthases, reinforcing the remarkable conservation of HMTs' function in metal detoxification.

### The N-terminal Extension Domain (NTE) is Essential, but not Sufficient for HMT-1-HMT-1 Interactions

Since the N-terminal extension domain (NTE) is a conserved structural feature of HMT-1 proteins, unique to this half-transporter family, we expected that it may be critical for HMT-1 function. Therefore, we used the mbSUS approach to test if deletion of the NTE domain would affect the ability of HMT-1 to interact with itself. Truncated HMT-1, lacking NTE (designated ΔNTE) or possessing NTE only (designated NTE), were fused at their C-termini with NubG of pXNgate21-3HA vector (ΔNTE-NubG, NTE-NubG, respectively, Fig. 3A) and co-expressed with HMT-1-CubPLV as described above. Interactions were assayed by monitoring colony formation of serially-diluted cell inocula on SC medium lacking adenine and histidine, or by *β-*galactosidase activity. Interactions were suppressed by supplementing SC medium with methionine (Fig. 3C).

We found that regardless of the approaches used for the analysis, interactions occurred only when a full-length HMT-1-NubG or -CubPLV fusions were co-expressed in diploid yeast cells. Interactions were significantly suppressed when a full-length HMT-1-CubPLV was co-expressed with HMT-1-NubG lacking NTE (Fig. 3C). Minimal, methionine-repressible cell growth relative to the negative control (HMT-1-CubPLV + *NubG*) was observed when ΔNTE-NubG prey construct was co-expressed with the full-length HMT-1-CubPLV bait. However, the *β*-galactosidase activity was detectible only when a full-length bait and prey constructs were co-expressed (Fig. 3C). Cell growth and the *β*-galactosidase activity assays can be interpreted to mean that the full-length HMT-1 and HMT-1 lacking NTE do not interact, or that interactions are very weak and are at the limit of detection.

**Figure 4:**
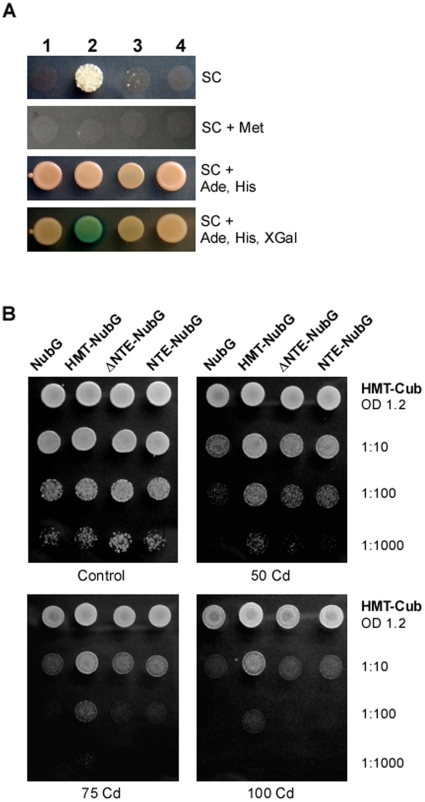
NTE is essential, but not sufficient for the ability of HMT-1 to confer Cd tolerance. **A.** mbSUS analysis of ΔHMT-1 self-association. Growth conditions are indicated on the left. Numbers across the top represent experiments and controls as follows: **1** - HMT-CubPLV + NubG; **2** – HMT-CubPLV + HMT-NubG; **3** - HMT-CubPLV + ΔNTE-NubG; **4** - ΔNTE-CubPLV + ΔNTE-NubG. Note the presence of cell growth and *β*-galactosidase activity in cells co-expressing the full-length HMT-1-NubG with HMT-1-CubPLV, but not ΔNTE-NubG with ΔNTE-CubPLV, or the full-length HMT-1-CubPLV with ΔNTE-NubG, or NubG only **B**. Dilution series of THY.AP5 cells expressing the proteins indicated at the top of the figure grown at the indicated concentrations of CdCl_2_. See legend to Figure 3 for the identity of the constructs. Concentrations of CdCl_2_ in µM are indicated below each experiment. Note the striking growth difference at 75 and 100 µM CdCl_2_.

Failure to detect HMT-1 self-association was not due to decreased expression or stability of the truncated HMT-1 since it was detected in microsomal membranes by immunoblot analysis (Fig. 3B). However, it was possible that the lack of interactions was due to fact that the NTE domain was necessary for targeting, but not for interactions. Therefore, HMT-1 without NTE and full-length HMT-1 might localize to different subcellular compartments. If the latter suggestion is correct, and NTE is dispensable for interactions, than co-expressed in yeast ΔNTE-NubG and ΔNTE-CubPLV constructs might interact since both would be in the same subcellular compartment. However, the interactions occurred only in cells co-expressing a full-length HMT-1-NubG or -CubPLV fusions, but not in cells co-expressing HMT-1-CubPLV and HMT-1-NubG constructs lacking NTE (Fig. 4A). These results further support our suggestion that the NTE domain is necessary for interactions, but do not rule out the possibility that it is also important for targeting.

Interactions were not observed when a full-length HMT-1-CubPLV was co-expressed with NTE-NubG construct (Fig. 3C). Failure to detect HMT-1 self-association was not due to decreased expression or stability of the NTE domain since it was detected in microsomal membranes by immunoblot analysis (Fig. 3B).

Since the NTE alone did not interact with the full-length HMT-1, we concluded that the NTE domain is not sufficient for HMT-1-HMT-1 interaction, and that other structural feature(s) must associate to form a functional transporter.

### NTE and Oligomerization are Essential for HMT-1 Function in Cd Detoxification

Since formation of at least a four-domain structure is a pre-requisite for the activity of half-molecule ABC transporters [13], [14], [16], [21], and truncated HMT-1 does not self-associate, we expected that truncated HMT-1 would not confer Cd tolerance in *S. cerevisiae*. Consistent with this prediction, the *C. elegans* HMT-1 deletion mutant, *gk155*, lacks two cytosolic loops and a transmembrane domain of the NTE and is hypersensitive to heavy metals [7].

To test our hypothesis we compared Cd sensitivity of THY.AP5 yeast expressing the empty pNXgate32/33-3HA (NubG) vector, the full-length HMT-1-NubG, the HMT-1-NubG lacking the NTE domain, or the NTE domain fused to NubG. Cd sensitivity was monitored as described above. We found that only the full-length HMT-1 conferred Cd tolerance of yeast cells (Fig. 4B). Since only a full-length HMT-1 is capable of interacting with itself in mbSUS (Fig. 3C), we concluded that self-association is essential for HMT-1 functional activity and that the NTE domain plays a structural role, preserving HMT-1 self-association and function in metal detoxification.

### Concluding Remarks

HMTs are acutely required for heavy metal detoxification in different species, act by unknown mechanisms, and are distinct from other ABC transporters due to their unique topology: they are half-molecule ABC proteins, and are the only half-transporters with an NTE. Since functional activity of half-molecule ABC transporters requires homo- and/or hetero-oligomerization, in this manuscript we addressed two basic questions. *First*, does functional HMT-1 oligomerize? *Second*, what is the role of the NTE domain in HMT-1 function? In this manuscript we present the following observations: *first*, HMT-1 exists in a protein complex in *C. elegans* and, as determined by mbSUS, at least homodimerizes in *S. cerevisiae*. Accordingly, the molecular mass of 269 kDa of PFO-extracted HMT-1-GFP, detected by gel-filtration chromatography, is consistent with its being a dimer (Fig. 1B,C). Nevertheless, since higher molecular mass species were also detected (*e.g.* 340 kDa, Fig. 1B, C), it is also possible that HMT-1 trimerizes, or that HMT-1 homodimers associate with other cellular protein(s). Future studies will discriminate between these possibilities and will establish the identity of proteins present in the HMT-1-associated protein complex. *Second*, the NTE domain plays a structural role and is necessary, but is not sufficient for HMT-1 homomerization, suggesting that other structural components must associate with HMT-1 to form a functional transporter. *Third*, the ability of HMT-1 to homomerize is needed for its function in Cd detoxification. Since the presence of NTE and heavy metal detoxification function of HMTs are conserved across species, we speculate that in addition to serving a structural role, the NTE domain possesses transport activity, and directly contributes to heavy metal detoxification.

The functional significance of the NTE has been studied in some full-molecule ABC transporters of the ABCC subfamily [22], [28], [29]. For instance, the NTE domain of a full-molecule ABC transporter, *S. cerevisiae* YCF1, is needed for vacuolar membrane trafficking and transport [28]. In contrast, the NTE domain of ABCC1/MRP1 has redundant trafficking signals with the COOH-region [29], but regulates its homodimerization [22]. Unlike CeHMT-1, the NTE of ABCC1/MRP1 is both essential and sufficient for oligomerization. We do not know whether the NTE domain of CeHMT-1 is required only for self-association or, as shown for full-molecule ABC transporters of ABCC subfamily, is also involved in membrane trafficking and transport activities. The prominence of heavy metals as environmental toxins and the remarkable conservation of HMT-1 structural architecture and function in different species reinforce the value of continued studies of HMT-1 in model systems for identifying functional domains in HMTs of humans.

## Materials and Methods

### *C. elegans* Strains and Growth Conditions

*C. elegans* strains used in this study are listed in Table 1. Worms were maintained at 20°C on solid Nematode Growth medium (NGM) using the *E. coli* OP50 strain as a food source [30].

**Table 1:** List of *C. elegans* strains.

Strain Name	Genotype	Source
N2	*C. elegans* wild type, *var*. Bristol	CGC
DP38	*unc-119(ed3)III*	CGC
VF1	*unc-119(ed3)III;gfEx1[phmt-1::GFP;unc-119(+)]*	[7]
VF3	*hmt-1(gk161)III*	[7]
DP38	*unc-119(ed3)III*	CGC
VF10	*unc-119 (ed3)III;gfEx2[phmt-1-hmt-1::GFP, unc-119(+)]*	This study
VF11	*unc-119(ed3)III;gfIs1[phmt-1-hmt-1::GFP, unc-119(+)]*	This study
VF12	*hmt-1(gk161)III;gfIs1[phmt-1-hmt-1::GFP, unc-119(+)]*	This study

### *S. cerevisiae* Strains and Growth Condition

THY.AP4 (*MATa leu2-3,112 ura3-52 trp1-289 lexA::HIS3 lexA::ADE2 lexA::lacZ*) and THY.AP5 (*MATα URA3 leu2-3,112 trp1-289 his3-Δ1 ade2Δ::loxP*) were obtained from the Dr. Wolf B. Frommer lab (Stanford University) depository at *Arabidopsis* Biological Resource Center (ABRC) http://www.arabidopsis.org/abrc/index.jsp. Yeast were cultured at 30°C on YPAD media [26], [27]. Yeast cells were transformed with bait and prey constructs using the LiOAc/polyethylene glycol method [31]. Transformants were selected for leucine or tryptophan prototrophy on synthetic complete (SC) media as described below and in [26], [27]. For evaluation of cadmium tolerance, the SC media was supplemented with CdCl_2_ at the indicated concentrations.

### Generation of Transgenic Worms Expressing HMT-1::GFP

A ten kb genomic fragment, consisting of the promoter (*phmt-1*) and genomic sequence of *C. elegans hmt-1*, was PCR-amplified and fused at the C-terminus of the translated polypeptide with GFP of the pPD117.01 vector [32]. Transgenic animals expressing HMT-1::GFP were generated by co-injecting the engineered construct (80 ng/µl) and the selectable marker, a plasmid carrying a functional gene (*unc-119^+^*, 100 ng/µl) into the gonadal syncytium of severely paralyzed, uncoordinated (uncoordinated, Unc) *unc-119(ed-3)* adult hermaphrodites [33], [34]. Non-Unc transgenic animals exhibiting GFP-mediated fluorescence were selected using a Leica MZ16FA automated fluorescence stereozoom microscope with a Leica EL6000 metal halide illuminator as described previously [7]. Given that DNA introduced into the germline *via* micro-injection rarely integrates into chromosomes, but generally is organized into extrachromosomal DNA arrays that are frequently lost during mitosis, we integrated HMT-1::GFP of one line, VF10.1, by γ-ray-induced integration and isolated eighteen independently-derived stable transgenic lines showing the same GFP expression pattern [34]. One of the resulting worm lines (VF11.1) was crossed into *hmt-1(gk161)* [7] to generate the *hmt-1* mutant strain (VF12) expressing HMT-1::GFP under the control of *hmt-1* promoter *(hmt-1(gk161)III;gfIs1[phmt-1-hmt-1::GFP,unc-119^+^])*. Since *hmt-1::GFP* rescued the Cd sensitivity of *hmt-1(161)* allele (not shown), we concluded that the construct is functional. Therefore, we used VF12 strain in subsequent studies.

### Preparation of microsomal and soluble proteins from *C. elegans*

Worms (VF1 and VF12 strains, expressing transcriptional *phmt-1::GFP* [7] or translational *phmt-1-hmt-1::GFP* constructs) were cultured at 20°C on NGM agar plates seeded with *Escherichia coli* OP50. Age-synchronized (young adults) worms were used for protein isolation and fractionation [35]. To generate sufficient age-synchronized worms, 60 young adults were placed per each of four 150×15 mm NGM agar plates with OP50, and cultured at standard condition for 3.5 days (until the progeny of inoculated worm were egg-laying adults and sufficient embryos were visible on the plates).

Young adult hermaphrodites were collected from plates with M9 medium and washed free from *E. coli* OP50 by two rounds of centrifugation (3,500× *g* for 2 min) and resuspension in M9 medium. To replace M9 medium with lysis buffer, the worm pellet from the second centrifugation was resuspended in lysis buffer containing 50 mM TRIS-HCl, pH 7.6, 2 mM 2-mercaptoethanol, 1 mM phenylmethylsulfonyl fluoride (PMSF), and 1 µg/ml each of leupeptin, aprotinin, and pepstatin. After centrifugation at 3,500× *g* for 2 min, the final worm pellet was resuspended in the same lysis buffer (1/1.5 of V worms/V buffer ratio) and transferred into eppendorf tubes. Worms were broken by sonication at 4°C in lysis buffer and worm debris was cleared by low-speed centrifugation at 3,500× *g* for 10 min. The supernatant, containing microsomal and soluble proteins, was collected and subjected to ultracentrifugation at 115,000× *g* for 1 h using a Beckman bench-top ultracentrifuge. The supernatant, containing soluble proteins was collected, frozen in liquid N2 and kept at −80°C for subsequent studies. The microsomal pellet (membrane-bound vesicles of total cellular membranes), was washed, re-pelleted at 115,000× *g*, resuspended in the same lysis buffer, frozen in liquid N2 and kept at −80°C for subsequent studies.

### Gel-filtration FPLC

Membrane proteins isolated from VF12 strains were solubilized prior to gel-filtration chromatography using either non-denaturing detergent perfluorooctanoate (PFO), which preserves interactions within protein oligomers [23], [24], or with SDS, a strong ionic detergent which disrupts protein interactions [25]. Briefly, aliquots of membrane proteins (150 µg) were suspended in a buffer containing 50 mM Tris-HCl, pH 7.4, 150 mM NaCl and PFO or SDS at a final concentration of 4% or 1%, respectively, and solubilized for 1 h at room temperature. The insoluble material was cleared by centrifugation at 11,000× *g* at 4°C for 10 min before 500 µl aliquots of the supernatants were injected onto Superose 6HR column (GE Healthcare) equilibrated with 50 mM Tris-HCl, pH 7.4, 150 mM NaCl and PFO (0.5%) or SDS (0.1%). The column was developed with the same buffer at a flow rate of 0.3 ml/min, 250 µl fractions were collected and proteins were precipitated with trichloracetic acid (TCA, to a final concentration of 10%) at 4°C overnight. After collecting the precipitated proteins by centrifugation at 11,000× *g* at 4°C for 10 min, proteins were washed-free from TCA by 3 rounds of resuspension with ethanol and centrifugation at 11,000× *g* at 4°C for 10 min. The protein pellet was air-dried and reconstituted in 100 mM Tris-HCl (pH 8.0). The distribution of HMT-1::GFP in collected fractions was then analyzed by SDS-PAGE and immunoblot analysis. The elution profiles of protein markers, including ovalbumin (45 kDa), conalbumin (75 kDa), aldolase (158 kDa), ferritin (440 kDa) and thyroglobulin (669 kDa) (GE Healthcare) were analyzed and detected in collected fractions using the UV detector of the FPLC system (AKTA purifier, GE Healthcare).

### Construction of HMT-1 Split-Ubiquitin Plasmids and Detection of HMT-1 Protein Interactions Using Mating-Based Split-Ubiquitin System (mbSUS)

The homomerization of HMT-1 was tested using the mbSUS approach [26], [27]. The NubG and CubPLV vectors, KAT1-CubPLV and NubG-KAT1 constructs and THY.AP4 and THY.AP5 were obtained from the Dr. Wolf B. Frommer lab (Stanford University) depository at Arabidopsis Biological Resource Center (ABRC) http://www.arabidopsis.org/abrc/index.jsp. The cDNAs corresponding to the full-length open reading frame (ORF) and partial cDNAs were amplified from *C. elegans* N2 strain RNA by reverse transcription (RT) PCR to generate constructs by *in vivo* cloning in yeast [26], [27]. Briefly, cDNAs were flanked with B1 and B2 linkers by PCR using the following primer pairs (Table 2). For NubG fusions, pNXgate32/33-3HA or pXNgate21-3HA vectors were cleaved with *EcoR*I/*Sma*I, whereas pMetYCgate vector was cleaved with *Pst*I/*Hin*dIII. Gel-purified PCR products and linearized vectors were co-transformed into THY.AP4 or THY.AP5 strains for creating CubPLV bait and NubG prey clones, respectively. THY.AP4 cells expressing CubPLV bait constructs were selected on SC medium for leucine prototrophy, whereas THY.AP5 cells expressing NubG-prey constructs were selected for tryptophan prototrophy. Several clones from each THY.AP5 and THY.AP4 transformation were incubated on appropriate SC medium with or without G418. Plasmids were extracted from cultures grown without G418 (cells carrying vectors with inserts did not grow on G418) and inserts were sequenced. THY.AP4 and THY.AP5 cells carrying bait or prey constructs, respectively, were mated and diploids, co-expressing bait and prey, were selected on SC medium lacking leucine, tryptophan and uracil, but containing adenine and histidine. Interactions were selected in diploid cells on SC medium lacking adenine and histidine. Interactions were suppressed by methionine (75 µM) [26], [27]. Growth was monitored for 2–9 days. Interactions were also verified using *β*-galactosidase assays as described [26], [27].

**Table 2:** The primer sequences used to clone the full-length and truncated CeHMT-1.

Name	Sequence (5′→3′)	Cloning Vector
*hmt-1* full-length-FW	ACAAGTTTGTACAAAAAAGCAGGCTCTCCAACCACCATGGGCTTT TCACCATTTCTCGA	mbSUS
*hmt-1* full-length-RV	TCCGCCACCACCAACCACTTTGTACAAGAAAGCTGGGTACGGAAGCTCCTCGCCGAGTTCAA	mbSUS
ΔNTE-FW	ACA AGT TTG TAC AAAAAA GCAGGCTCTCCAACCACCATGCAA CTT CGC GTC GTT TTT TG	mbSUS
ΔNTE-RV	TCCGCCACCACCAACCACTTTGTACAAGAAAGCTGGGTACGGAAGCTCCTCGCCGAGTTCAA	mbSUS
NTE-FW	ACAAGTTTGTACAAAAAAGCAGGCTCTCCAACCACCATGGGCTTT TCACCATTTCTCGA	mbSUS
NTE-RV	TCCGCCACCACCAACCACTTTGTACAAGAAAGCTGGGTAGAGGGAAATTGATTTTGTCG	mbSUS
*phmt-1*-FW	CCCAGGGGCCGCGGAAACTAGTTTTTTAAATTAATAAATT	pPD117.01
*phmt-1*-RV	CTTATAAGGTACCGGAAGCTCCTCGCCGAGTTCAATCG	pPD117.01

### Subcellular Fractionation of *S. cerevisiae*

Membrane proteins were isolated and fractionated using modified procedures [36], [37]. Briefly, *S. cerevisiae* THY.AP5 cells expressing different HMT-1-NubG-fused constructs, or the empty NubG vector were converted to spheroplasts with Zymolyase 20T (ICN) in a buffer that also contained 1% (w/v) yeast extract, 2% (w/v) Bacto-Peptone) 0.7 M sorbitol, 1% (w/v) dextrose, 5 mM dithiothreitol, and 100 mM Tris-Mes (pH 7.5). Spheroplasts were disrupted by homogenization in a Dounce homogenizer in a medium containing 50 mM Tris-HCl, pH 7.6, 2 mM dithiothreitol, 1 mM EGTA, 1 mM phenylmethyl sulfonylfluoride and 1 µg/ml each of leupeptin, pepstatin, and aprotinin. The crude lysate was cleared by centrifugation at 4,000× *g* for 10 min. The lysate was then spun at 100,000× *g* for 30 min to pellet total microsomal membranes. Membranes were reconstituted in the same buffer containing 10% glycerol, frozen in liquid nitrogen and stored at −80°C.

### SDS-PAGE and Western Blot Analyses

Aliquots of proteins (30 µg/lane) were subjected to SDS-PAGE on 7% (w/v) gels and electrotransferred to nitrocellulose filters for 18 h at 4°C at a constant current of 60 mA in Towbin buffer containing 0.05% SDS [38]. For immunodetection of GFP epitope, the nitrocellulose blots were probed with the primary goat polyclonal anti-GFP antibody (1∶1,000 dilution, Rockland Immunochemicals), and with the secondary HP-conjugated anti-goat IgG antibody (1∶2,500, Rockland Immunochemicals). For immunodetection of influenza hemagglutinin-HA epitope, microsomal membrane proteins were de-lipidated in TCA prior to SDS-PAGE and immunoblot analysis [39]. The nitrocellulose blots were probed with the primary rabbit polyclonal anti-HA antibody (1∶2,000 dilution, Sigma) and secondary, an HR-conjugated anti-rabbit IgG antibody (1∶10,000 dilution GE Healthcare). In both cases, immunoreactive bands were visualized with ECL using the LumiGLO system (KPL).

### Protein Estimation

Soluble proteins were estimated by a dye-binding method [40]. To estimate membrane proteins, a dye-binding method was modified to solubilized proteins in 0.2% Triton-X100.
